# The role of ^18^F-FDG-PET/CT in evaluating retroperitoneal masses -Keeping your eye on the ball!

**DOI:** 10.1186/s40644-019-0217-5

**Published:** 2019-05-29

**Authors:** Te-Jui Hung, Luke McLean, Catherine Mitchell, Claire Pascoe, Nathan Lawrentschuk, Declan G. Murphy, Amir Iravani, Dalveer Singh, Michael S. Hofman, Lamiaa Zidan, Tim Akhurst, Jeremy Lewin, Rodney J. Hicks

**Affiliations:** 1 Cancer Imaging, Peter MacCallum Cancer Centre, Melbourne, Australia; 2 Department of Medical Oncology, Peter MacCallum Cancer Centre, Melbourne, Australia; 3 Department of Pathology, Peter MacCallum Cancer Centre, Melbourne, Australia; 4 Division of Cancer Surgery, Peter MacCallum Cancer Centre, Melbourne, Australia; 5 ONTrac at Peter Mac Victoria Adolescent and Young Adult Cancer Centre, Peter MacCallum Cancer Centre, Melbourne, Australia; 6 Sir Peter MacCallum Department of Oncology, University of Melbourne, Parkville, Australia

**Keywords:** FDG-PET/CT, Seminoma, Germ cell tumour, Cryptorchidism, Retroperitoneal

## Abstract

**Background:**

Testicular germ cell tumour is the commonest malignancy affecting males aged between 15 and 35, with an increased relative risk amongst those with a history of cryptorchidism. In patients presenting with locoregional metastatic disease, retroperitoneal and pelvic soft tissue masses are common findings on ultrasound and computed tomography, which has several differential diagnoses within this demographic cohort. On staging ^18^F-FDG-PET/CT, understanding the typical testicular lymphatic drainage pathway facilitates prompt recognition of the pathognomonic constellation of unilateral absence of testicular scrotal activity, and FDG-avid nodal masses along the drainage pathway. We describe the cases of three young males presenting with abdominopelvic masses, in whom FDG-PET/CT was helpful in formulating a unifying diagnosis of metastatic seminoma, retrospectively corroborated by a history of testicular maldescent.

**Case presentations:**

In all three cases, the patients were males aged in their 30s and 40s who were brought to medical attention for back and lower abdominal pain of varying duration. Initial imaging evaluation with computed tomography and/or ultrasound revealed large abdominopelvic soft tissue masses, with lymphoproliferative disorders or soft tissue sarcomas being high on the list of differential diagnoses.

As such, they were referred for staging FDG-PET/CT, all of whom demonstrated the pathognomonic constellation of, 1) unilateral absence of scrotal testicular activity, and 2) FDG-avid nodal masses along the typical testicular lymphatic drainage pathway. These characteristic patterns were corroborated by a targeted clinical history and examination which revealed a history of cryptorchidism, and elevated β-hCG in two of three patients. All were subsequently confirmed as metastatic seminoma on biopsy and open resection.

**Conclusion:**

These cases highlight the importance of clinical history and examination for the clinician, as well as a sound knowledge of the typical testicular lymphatic drainage pathway for the PET physician, which would assist with prompt recognition of the characteristic imaging patterns on FDG-PET/CT. It further anecdotally supports the utility of FDG-PET/CT in evaluating undiagnosed abdominopelvic masses, as well as a potential role in the initial staging of germ cell tumours in appropriately selected patients.

## Background

Testicular germ cell tumours (GCT) are the commonest malignancy amongst young males aged 15–35 [1]. Survival outcomes have significantly improved over the past four decades across all stages of disease with the introduction of platinum-based chemotherapy, achieving a five-year survival over 90% in patients with good-risk disease [2–4]. The association between cryptorchidism and testicular GCT is well recognised with a relative risk of 3.7–7.5 times higher than the scrotal testis population. Testicular maldescent is identified in a significant proportion of men who develop testicular cancer, and undescended and retractile testicles represent an important risk factor in the development of germ cell tumours being associated with a 2–8 times increased risk of malignancy [5–9].

Accurate staging is essential in facilitating the optimal treatment plan. Contrast-enhanced computed tomography (CT) +/− magnetic resonance imaging (MRI) of the brain in selected patients remain the recommended imaging modalities of choice for initial staging by the European Society of Medical Oncology and National Comprehensive Cancer Network. The currently recommended role of ^18^F- fluorodeoxyglucose positron emission tomography/computed tomography (FDG-PET/CT) in the management of GCT tumours is limited to evaluating residual masses > 3 cm in the post-treatment setting in pure seminoma, however there may be a role for FDG-PET/CT in assessing equivocal CT findings [11–13].

In recent years, FDG-PET/CT has become an important modality in oncologic imaging, with high management impact reported across numerous tumour streams, particularly in characterizing masses of uncertain aetiology [14]. We present three cases of retroperitoneal masses for which staging FDG-PET/CT was helpful in establishing a unifying diagnosis of metastatic seminoma.

## Case presentation

### Case one

**Fig. 1:**
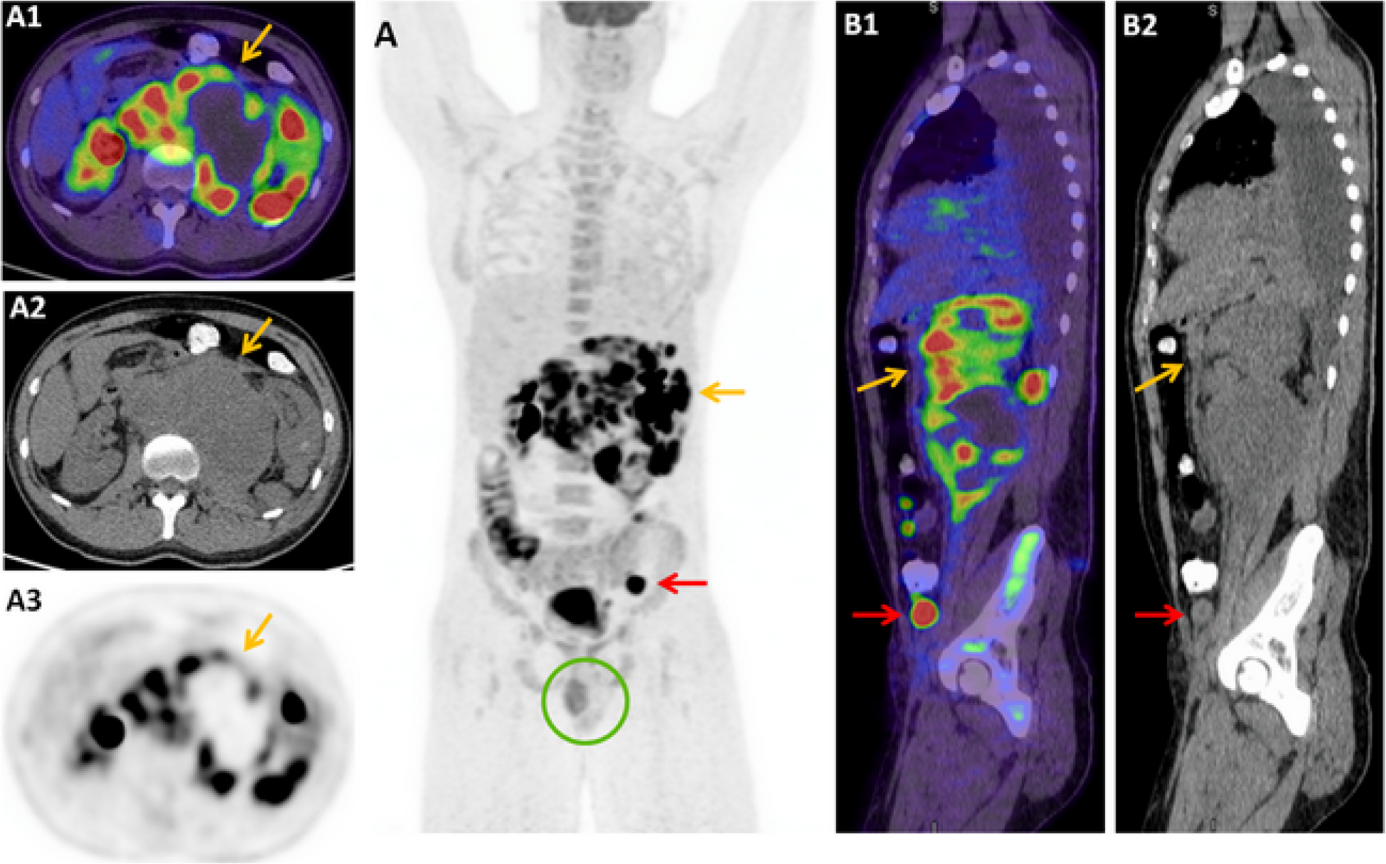
Case One - Staging FDG-PET/CT demonstrates intense FDG-avidity (SUVmax 19) in a bulky and heterogeneous retroperitoneal mass [*orange arrows*] (**a**1–3, **b**1–2). A retractile testis [*red arrow*] (**b**1–2), also intensely FDG-avid (SUVmax 16.4), is shown to be undescended at the time of imaging, situated adjacent to the deep inguinal ring. The maximal intensity projection (MIP) image (**a**) demonstrates absent left scrotal testicular activity [*circle*]

A 30-year-old male presented with a six-month history of lower back pain and four weeks of fevers. CT abdomen/pelvis demonstrated a bulky retroperitoneal mass with bilateral retroperitoneal and left pelvic lymphadenopathy, and numerous omental deposits. He was subsequently referred to the Haematology Service at our institution with a presumptive diagnosis of lymphoma. Staging FDG-PET/CT demonstrated an intensely FDG-avid and bulky left retroperitoneal mass causing lateral displacement of the left kidney, with a maximum standardised uptake value (SUVmax) of 19. Numerous FDG-avid soft tissue deposits were seen in the bilateral retroperitoneal space. The left testis was notably absent in the scrotal sac, with an intensely FDG-avid (SUVmax 16.3) soft tissue lesion situated adjacent to the left inguinal canal, which had been presumed to reflect lymphomatous involvement of an external iliac node on prior CT (Fig. 1). The constellation of absent left testicular activity and intensely FDG-avid retroperitoneal mass raised the prospect of metastatic seminoma, which was subsequently supported biochemically with a mildly elevated β-human chorionic gonadotrophin (β-hCG) at 56.3 IU/L (normal range < 2.6 IU/L); α-fetoprotein (AFP) was within the normal range. A bone marrow biopsy performed prior to the FDG-PET/CT was later found to reveal a normocellular marrow with no evidence of neoplastic involvement.

**Fig. 2:**
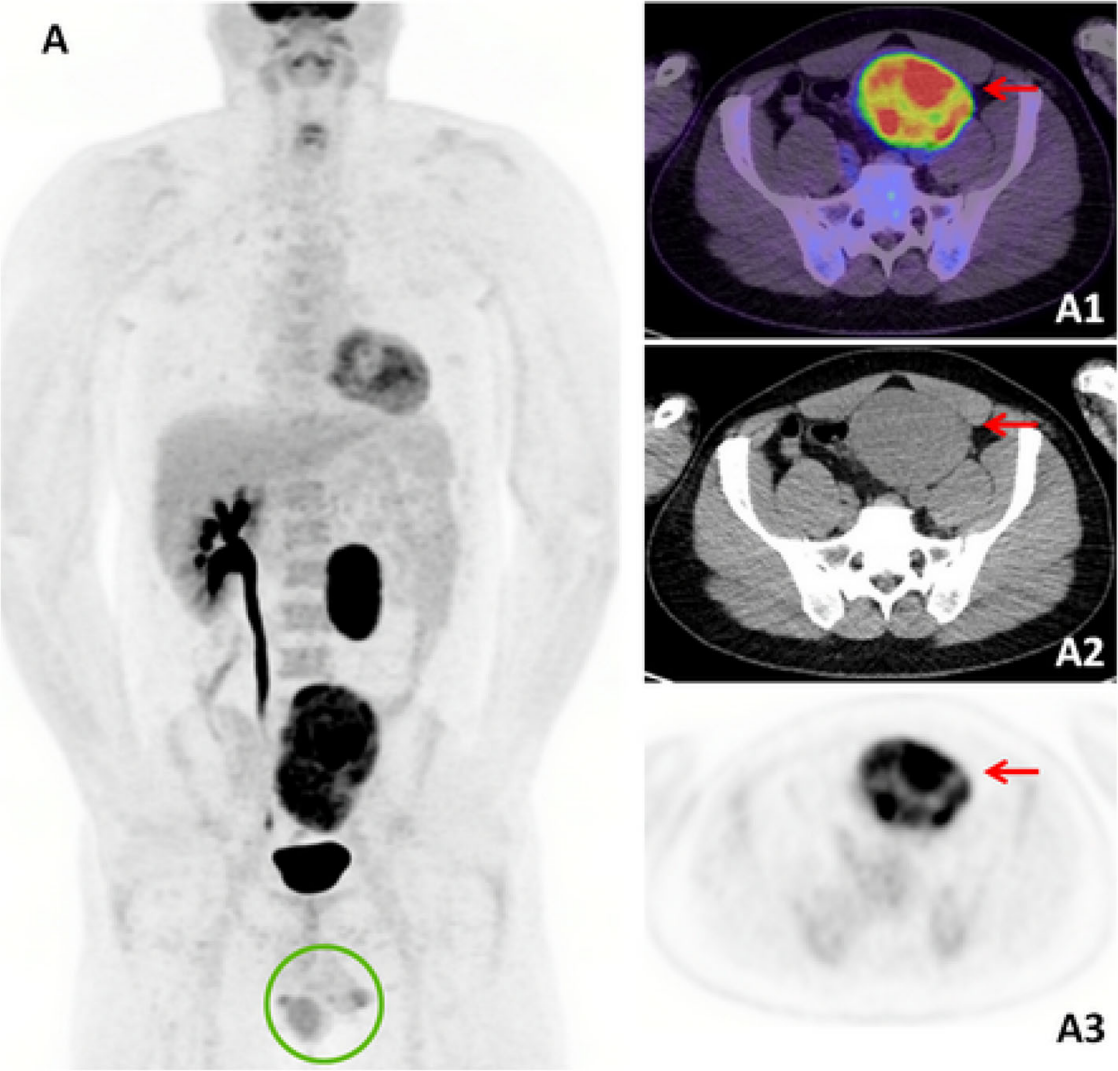
Case two - FDG-PET/CT maximum intensity projection (MIP) image demonstrates two large FDG-avid abdominopelvic masses, with absent left testicular scrotal activity [*circle*] (**a**). The dominant pelvic mass exhibits intense FDG-avidity (SUVmax 18.7) [*red arrow*]

Interestingly, a targeted examination was incongruent with the FDG-PET/CT findings, with a palpable left testicular mass and absent left inguinal lymphadenopathy. Scrotal ultrasound also demonstrated two testicles, with the 19 mm heterogeneously hypoechoic left testicular mass compatible with malignancy. On retrospective review of the FDG-PET/CT, metastatic testicular GCT arising from a retractile left testis was deemed to be the most likely unifying diagnosis (Fig. 2). Histological confirmation of seminoma was established following a CT-guided biopsy of the retroperitoneal mass, with immunohistochemistry positivity for CD117 (c-KIT) and Oct 3/4, and negative for cytokeratin AE1/AE3, S100 protein, CD45, desmin, and CD56. He was subsequently treated with Cisplatin-based chemotherapy.

### Case 2

**Fig. 3:**
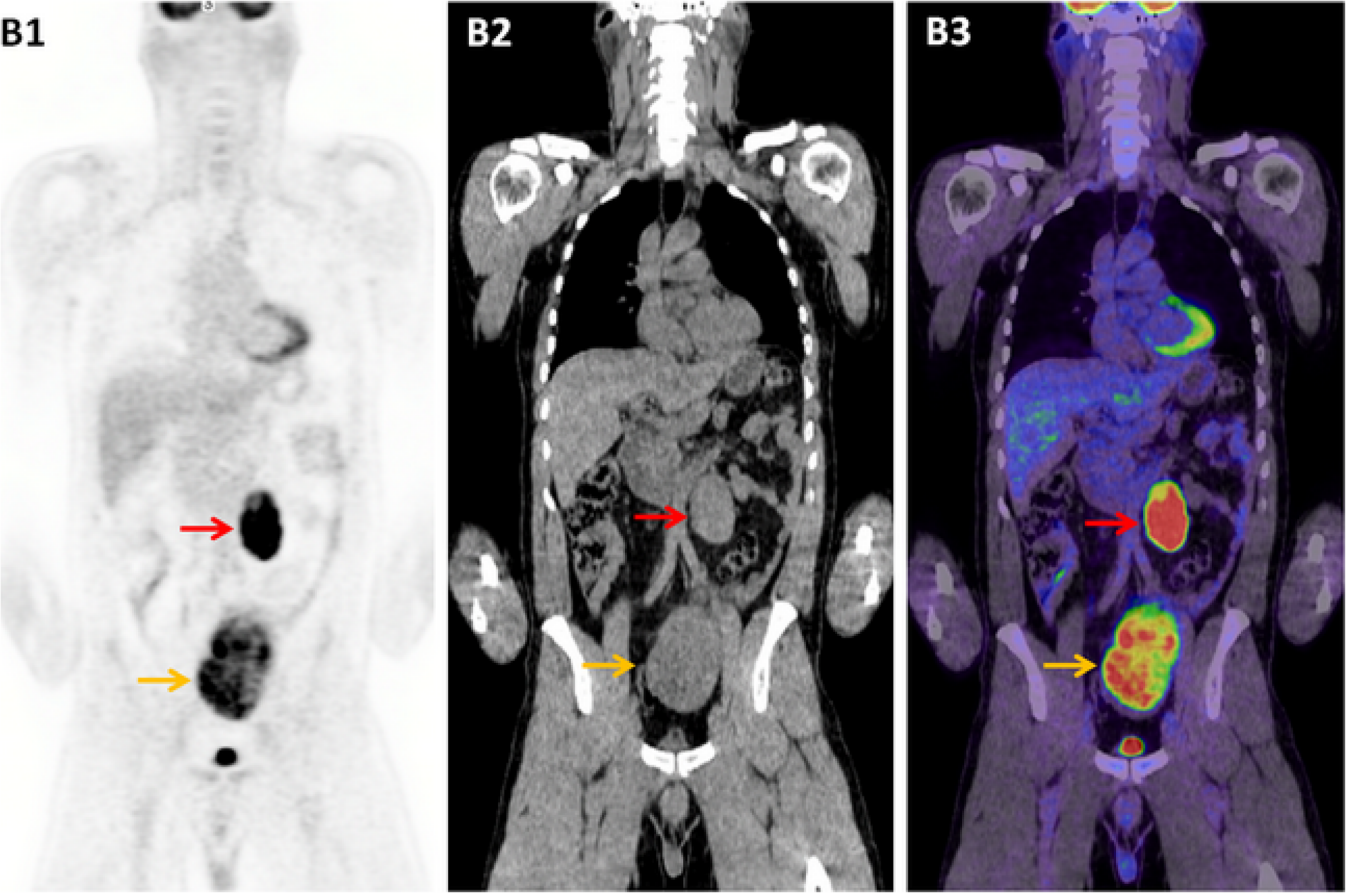
Case Two - The coronal view illustrates the two FDG-avid nodal masses following the typical male genitalia lymphatic drainage pathway, situated in the pelvic cavity [*orange arrow*] and the retroperitoneum [*red arrow*] (**b**1–3)

A 42-year-old male presented with a one-week history of lower abdominal pain. Abdominal ultrasound demonstrated two large abdominopelvic masses which were corroborated on contrast-enhanced CT chest/abdomen/pelvis. Serum LDH was mildly elevated at 267 U/L (normal range 120–250 U/L), and with lymphoma being the initial top differential diagnosis, a staging FDG-PET/CT was performed (Figs. 2 and 3). The left retroperitoneal mass lesion measured 4.5 × 4.3 × 7.7 cm in size and the left pelvic mass was 8.9 × 6.9 × 11.9 cm, with both masses exhibiting intense FDG-avidity (SUVmax 21.4). A solitary right testis was noted in the scrotal sac.

Further questioning revealed a background of undescended testis at birth which was subsequently resected in childhood. Metastatic GCT became the primary differential diagnosis, further supported biochemically with a mildly elevated β-hCG at 6.4 IU/L. Subsequent ultrasound-guided biopsy of the left pelvic mass confirmed metastatic seminoma. He was subsequently commenced on BEP chemotherapy.

### Case 3

**Fig. 4:**
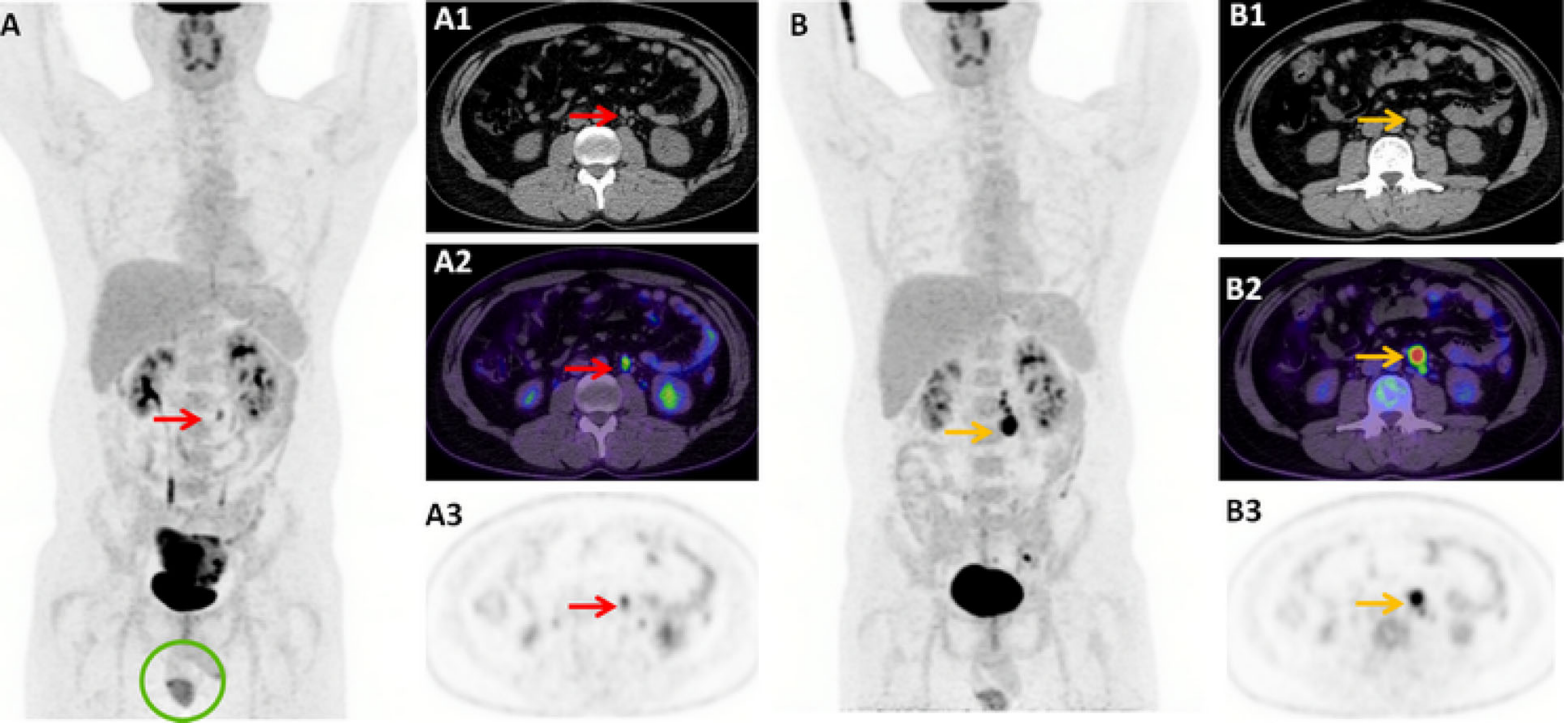
Case Three - Staging FDG-PET/CT demonstrates a bulky and intensely FDG-avid pelvic mass, with absent left scrotal testicular activity [*circle*] (**a**). A small focus of mild FDG-uptake (SUVmax 4.9) in the upper abdomen corresponds with a 10 mm left para-aortic node on the co-acquired non-contrast enhanced CT [*red arrow*] (**a**1–3). Re-staging ^18^F-FDG-PET/CT following pelvic resection revealed interval metabolic and structural progression of the left para-aortic node [*orange arrow*] (20 mm; SUVmax 13.4), with a chain of new FDG-avid subcentimetre nodes cranially (B1–3)

33-year-old male presented with lower abdominal pain. Contrast-enhanced CT abdomen and pelvis revealed a large 7.8 × 8.0 × 8.3 cm pelvic mass, initially thought to be a soft tissue sarcoma. A staging FDG-PET/CT was performed which demonstrated intense FDG-avidity (SUVmax 12.4) in relation to the pelvic mass lesion, a mildly FDG-avid (SUVmax 4.9) left para-aortic node deemed highly suspicious for a retroperitoneal nodal metastasis, and, notably, an absent left testis (Fig. 4).

**Fig. 5:**
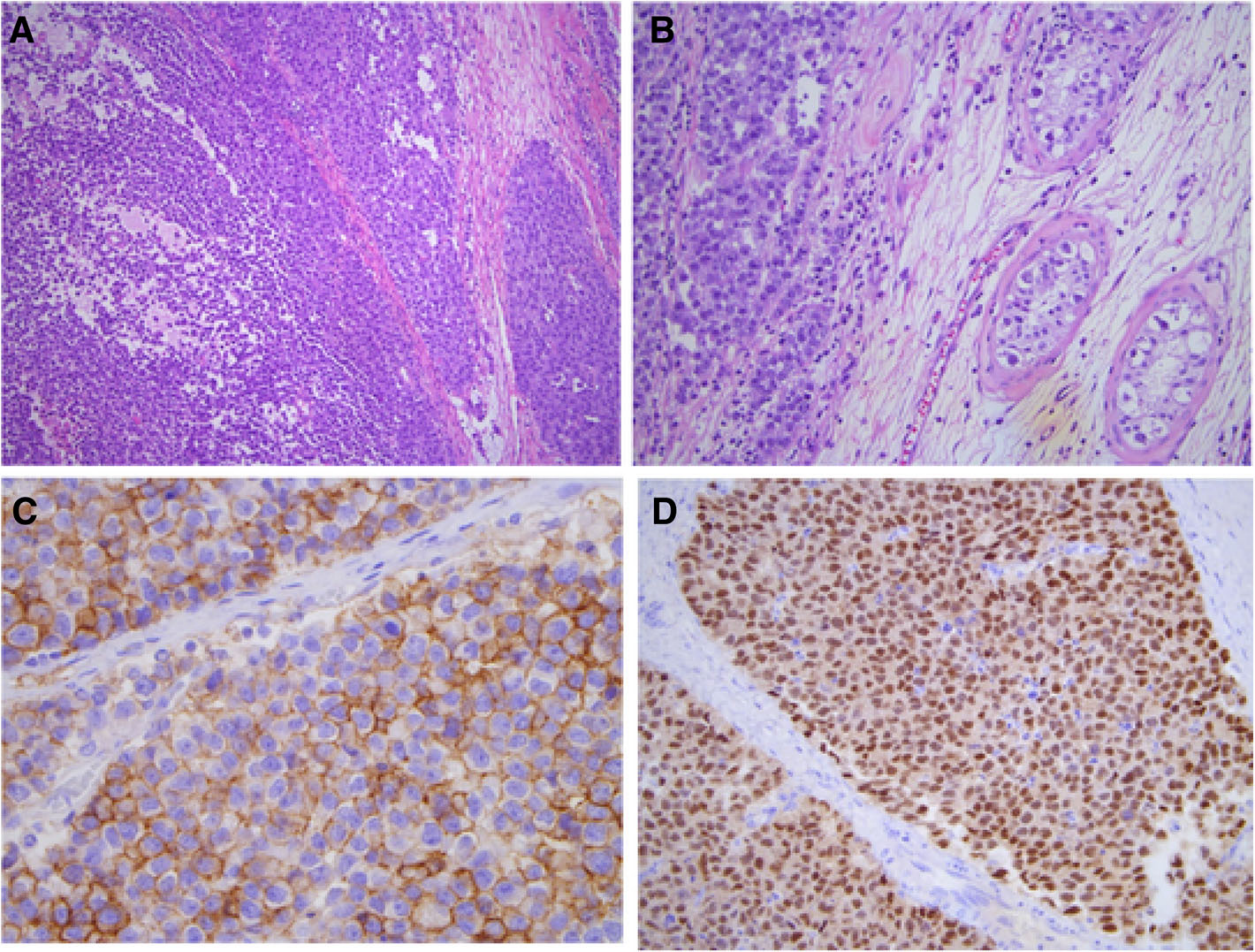
Case Three - Histopathological examination of the intra-abdominal mass showed seminoma, characterised by a monotonous population of polygonal cells (**a** Haematoxylin & Eosin (H&E), original magnification × 100). Focally, residual seminiferous tubules were seen, with germ cell neoplasia in situ (**b** H&E, original magnification × 200). Immunohistochemistry showed that the tumour cells were reactive for CD117 (**c** original magnification × 400) and Oct 3/4 (**d** original magnification × 200)

Further history included the patient describing an absent left testis from birth which was never brought to medical attention. CT-guided biopsy of the pelvic mass revealed a seminoma with normal tumour markers. Open resection of the pelvic mass confirmed it to be arising from an intra-abdominal testis, with focal residual seminiferous tubules evident and showing germ cell neoplasia in situ (Fig. 5). Restaging FDG-PET/CT performed 3 months post-surgical resection demonstrated interval progression of the pre-existing FDG-avid left para-aortic node, confirming metastatic involvement. He was subsequently commenced on BEP chemotherapy.

## Discussion

FDG-PET/CT has proven to be a powerful oncologic imaging modality, with high clinical impact reported in the settings of diagnosis, staging, response assessment, and surveillance across numerous cancer types [15]. In the management of testicular GCT, its role remains unclear although is currently limited to the evaluation of residual masses post-chemotherapy in patients with seminoma, and tumour detection in those with suspected recurrence and negative conventional imaging. Current society guidelines recommend CT as the imaging test of choice during the initial staging of patients with metastatic GCT, and MRI brain in those with high clinical suspicion of brain disease [11–13]. There may be a role for FDG-PET/CT in the evaluation of equivocal nodal findings on staging CT, however this requires further evaluation [11]. GCTs typically demonstrate high FDG-avidity, particularly in pure seminomas [16, 17], however, historically there has been little in the literature to support the use of FDG-PET/CT in initial disease staging. Several studies have explored the role of FDG-PET/CT in testicular GCTs, particularly in the staging of Stage I/II disease, but only two reported a higher sensitivity and negative predictive value over conventional CT [17–24]. One consistently reported limitation is the lack of sensitivity of FDG-PET/CT in detecting subcentimetre retroperitoneal nodal metastases. In the post-chemotherapy setting of pure seminomas, radiologically detectable residual masses are seen in approximately 50–80% of cases and many of these often represent fibrosis/necrosis rather than viable tumour. In this clinical context, there is conflicting evidence regarding the utility of FDG-PET/CT, especially pertaining to positive scans in patients with residual masses > 3 cm. There is general consensus that a negative FDG-PET/CT performed no earlier than six weeks following completion of chemotherapy is highly reassuring by virtue of a consistently reported high negative predictive value [25–27]. However, the positive predictive value has varied widely from 100% in the SEMPET trial [26], 58% in the meta-analysis by Treglia et al. [25], and 23% in a recent retrospective series by Cathomas et al. [27]. Post-treatment inflammatory change, tumour necrosis, and granulomatous change are recognised factors that can contribute to false-positive readings. This remains a controversial area and further prospective research is required to elucidate the role of positive FDG-PET/CT in guiding clinical management post-chemotherapy.

Whilst ~ 70% of cases of seminoma are diagnosed as stage I disease, for the 10% of patients with advanced metastatic disease requiring systemic therapy, establishing prompt and accurate diagnosis is not always straight forward as we have shown with our series. Patients within this demographic cohort often have numerous overlapping clinical and imaging characteristics, and presenting symptoms, with other differential diagnoses such as lymphoproliferative malignancies and soft tissue sarcoma. Tumour markers have been shown to have limited sensitivity in advanced seminoma with only 15–20% of patients having a mildly elevated β-hCG at diagnosis [28]. A unique strength of FDG-PET/CT lies in its ability to provide a whole-body metabolic evaluation. Indeed, standard CT body protocols in most institutions scan to the level of pubic symphysis or ischial tuberosities to avoid testicular irradiation. It is also not possible to be certain of the presence of complete spermatic cord structures on CT. In patients with metastatic disease Stage II or higher [10], having a sound understanding of the pathways of testicular lymphatic drainage is an important tool for diagnostic recognition. Testicular lymphatic drainage follows the testicular vein to the level of the renal vein, leading to retroperitoneal nodes at this level. As tumour volume increases, tumour may then spread caudally to involve pelvic nodes in the common iliac, internal iliac, and external iliac stations [29]. These characteristic patterns of tumour involvement are readily recognisable on the FDG-PET/CT maximal intensity projection (MIP) image. In case number three, small volume nodal disease was identified at the left renal hilum that was negative on staging CT but was subsequently confirmed to represent metastasis by virtue of local progression.

There have been numerous cases of metastatic seminoma arising from undescended testis described in the literature, describing young males aged in their 20s and 30s presenting with painless masses variably located in the abdominopelvic cavity and inguinal region [30–33]. In two of the cases, the patients underwent FDG-PET/CT as part of the staging process [30, 31] whilst the others were staged with conventional anatomical imaging and exploratory laparotomy. In our case series, the constellation of unilateral absence of scrotal testicular activity, and FDG-avid lymph nodes along the retroperitoneal and pelvic lymphatic pathways on FDG-PET/CT was pathognomonic and negated the prior presumed differential diagnoses of lymphoma and sarcoma. Recognition of these patterns could expedite formulating a unifying diagnosis and potentially minimise unnecessary and/or invasive investigations, which included a bone marrow biopsy in case number one.

## Conclusion

In these three cases of undiagnosed retroperitoneal masses, we describe the pathognomonic FDG-PET/CT findings of unilateral absence of scrotal testicular activity and intensely FDG-avid lymph nodes along the typical lymphatic drainage pathway that enabled imaging diagnosis of metastatic seminoma. In each case, this was associated with childhood testicular maldescent, emphasizing the importance of clinical history and examination in such cases. Recognition of these characteristic patterns facilitates prompt and accurate unifying diagnosis, minimise unnecessary investigations, and expedite appropriate treatment plans. These cases anecdotally support the use of FDG-PET/CT for initial disease staging in appropriately selected patients.

## Data Availability

The clinical and imaging data are available from the corresponding author upon request.

## Abbreviations

AFP: Alpha fetoprotein
BEP: Bleomycin-etoposide-platinum
CT: Computed tomography
FDG: Fluorodeoxyglucose
GCT: Germ cell tumour
LDH: Lactate dehydrogenase
MIP: Maximum-intensity projection
PET/CT: Positron Emission Tomography/Computed Tomography
SUV: Standardized uptake value
β-hCG: β-Human chorionic gonadotrophin

## References

[1] Djaladat H, Nichols CR, Daneshmand S (2011). Chemoresponsive liver hemangioma in a patient with a metastatic germ cell tumor. J Clin Oncol.

[2] Einhorn LH (1990). Treatment of testicular cancer: a new and improved model. J Clin Oncol.

[3] Einhorn LH, Williams SD, Chamness A, Brames MJ, Perkins SM, Abonour R (2007). High-dose chemotherapy and stem-cell rescue for metastatic germ-cell tumors. N Engl J Med.

[4] Bosl GJ, Motzer RJ (1997). Testicular germ-cell cancer. N Engl J Med.

[5] Pettersson A, Richiardi L, Nordenskjold A, Kaijser M, Akre O (2007). Age at surgery for undescended testis and risk of testicular cancer. N Engl J Med.

[6] Toppari J, Kaleva M (1999). Maldescendus testis. Horm Res.

[7] Dieckmann KP, Pichlmeier U (2004). Clinical epidemiology of testicular germ cell tumors. World J Urol.

[8] Thorup J, Mclachlan R, Cortes D, Nation T, Balic A (2010). What is new in cryptorchidism and hypospadias - a critical review on the testicular dysgenesis hypothesis. J Pediatr Surg.

[9] Ferguson L, Agoulnik AI (2013). Testicular cancer and cryptorchidism. Front Endocrinol.

[10] International Germ Cell Consensus Classification (1997). A prognostic factor-based staging system for metastatic germ cell cancers. International germ cell Cancer collaborative group. J Clin Oncol.

[11] Honecker F, Aparicio J, Berney D (2018). ESMO consensus conference on testicular germ cell cancer: diagnosis, treatment and follow up. Ann Oncol.

[12] National Comprehensice Cancer Network guidelines. Testicular Cancer (Version 1. 2019). http://www.nccn.org/professionals/physician_gls/pdf/testicular.pdf. Accessed 15 Mar 2019.

[13] Albers P, Albrecht W, Algaba F (2015). Guidelines on testicular cancer: 2015 update. Eur Urol.

[14] Hick RJ (2012). Should positron emission tomography/computed tomography be the first rather than the last test performed in the assessment of cancer?. Cancer Imaging.

[15] Hofman M, Hicks RJ. How we read oncologic FDG PET/CT. Cancer Imaging. 2016;16(1):35. 10.1186/s40644-016-0091-3 PMC5067887 27756360

[16] Bouchelouche K, Choyke PL (2015). PET/computed tomography in renal, bladder, and testicular cancer. PET Clin.

[17] Wilson CB, Young HE, Ott RJ, Flower MA, Cronin BF, Pratt BE (1995). Imaging metastatic testicular germ cell tumours with 18FDG positron emission tomography: prospects for detection and management. Eur J Nucl Med.

[18] Hofer C, Kubler H, Hartung R, Breul J, Avril N (2001). Diagnosis and monitoring of urological tumors using positron emission tomography. Eur Urol.

[19] Spermon JR, De Geus-Oei LF, Kiemeney LA, Witjes JA, Oyen WJ (2002). The role of (18)fluoro-2-deoxyglucose positron emission tomography in initial staging and re-staging after chemotherapy for testicular germ cell tumours. BJU Int.

[20] Tsatalpas P, Beuthien-Baumann B, Kropp J, Manseck A, Tiepolt C, Hakenberg OW (2002). Diagnostic value of 18F-FDG positron emission tomography for detection and treatment control of malignant germ cell tumors. Urol Int.

[21] Hain SF, O'Doherty MJ, Timothy AR, Leslie MD, Partridge SE, Huddart RA (2000). Fluorodeoxyglucose PET in the initial staging of germ cell tumours. Eur J Nucl Med.

[22] Albers P, Bender H, Yilmaz H, Schoeneich G, Biersack HJ, Mueller SC (1999). Positron emission tomography in the clinical staging of patients with stage I and II testicular germ cell tumors. Urology.

[23] Cook GJ, Sohaib A, Huddart RA (2015). The role of ^18^F-FDG PET/CT in the management of testicular cancers. Nucl Med Comm.

[24] de Wit M, Brenner W, Hartmann M (2008). 18F-FDG-PET in clinical stage I/II non-seminomatous germ cell tumours: results of the German multicentre trial. Ann Oncol.

[25] Treglia G, Sadeghi R, Annunziata S (2014). Diagnostic performance of fluorine-18-fluorodeoxyglucose positron emission tomography in the postchemotherapy management of patients with seminoma: systematic review and meta-analysis. Biomed Res Int.

[26] de Santis M, Becherer A, Bokemeyer C (2004). 2-^18^fluoro-deoxy-D-glucose positron emission tomography is a reliable predictor for viable tumor in postchemotherapy seminoma: an update of the prospective multicentric SEMPET trial. J Clin Oncol.

[27] Cathomas R, Klingbiel D, Bernard B (2018). Questioning the value of fluorodeoxyglucose positron emission tomography for residual lesions after chemotherapy for metastatic seminoma: results of an international global germ cell Cancer group registry. J Clin Oncol.

[28] Milowsky M. Genitourinary cancer. In: Hensley M, editor. ASCO-SEP 6th edition. Alexandria; 2018.

[29] Pano B, Sebastia C, Bunesch L (2011). Pathways of lymphatic spread in male urogenital pelvic malignancies. Radiographics.

[30] Bose S, Sengupta S, Mukherjee R (2017). Seminoma in an undescended testis. SM J Surg.

[31] Yilmaz A, Bayraktar B, Sagiroglu J, Gucluer B (2011). Giant seminoma in an undescended testis presenting as an abdominal wall mass. J Surg Case Rep.

[32] Althaf S, Shankar K, Kurpad V, Suma MN (2017). Seminoma of undesended testis with urinary bladder metastasis: a case report with review of literature. Urol Ann.

[33] Carlotto J, Colleoni-Neto R, Shigueoka D (2015). Intra-abdominal seminoma testis in adult: case report. Arq Bras Cir Dig.

